# Isochrony in titi monkeys duets: social context as a proximate cause of duets’ rhythm and regularity

**DOI:** 10.1098/rspb.2024.2805

**Published:** 2025-02-19

**Authors:** Chiara De Gregorio, Paola Antonini, Eckhard W. Heymann, Marco Gamba

**Affiliations:** ^1^ Department of Life Sciences and Systems Biology, University of Torino, Torino, Italy; ^2^ Department of Psychology, University of Warwick, Coventry, UK; ^3^ Behavioral Ecology and Sociobiology, German Primate Center, Leibniz Institute for Primate Research, Göttingen, Germany

**Keywords:** song, infant, singing primates, tempo, trade-off, coordination

## Abstract

Music and rhythm are typical features of all human cultures, but their biological origins remain unclear. Recent investigations suggest that rhythmic features of human music are shared with animal vocalizations. Moreover, arousal is known to influence the structure of both human speech and animal sounds. We investigated coppery titi monkeys’ (*Plecturocebus cupreus*) duet rhythms to assess adherence to rhythmic patterns previously observed only in Old World primates and to deepen our understanding of the proximate causes of non-human primate song rhythm. Titis’ songs were remarkably isochronous, but their *tempo* depended on the social context: songs sung during territorial confrontations have a slower pace than during early morning singing. Songs had a faster *tempo* and were less regular when infants were present, suggesting a speed-accuracy trade-off. Finally, we found that pair-mates perform isochronous songs with the same precision, suggesting that isochrony plays a role in boosting pair coordination, as it does in other singing primates. Our investigation sheds light on the ultimate and proximate causes of primates’ isochronous rhythm, to our knowledge confirming its presence for the first time in a New World monkey and highlighting the role of social factors in shaping its timing and regularity in the short term.

## 1. Introduction

Rhythm is everywhere in the biological world. Many animal behaviours are fundamentally periodic activities [1]. Physiological processes, such as heartbeat activity and respiration, occur rhythmically on small timescales [2]. By contrast, reproductive behaviour or sleeping patterns can occur seasonally or daily. Rhythm is also a fundamental characteristic of the vocalizations of both humans and many animals. Rhythm can be defined as a pattern of sounds and silences that iterate over time. Both human music and speech have a rhythmic structure [3–5], but the biological origins of rhythmic abilities in our and other species are unclear. In music, isochronous rhythms are the most common. Sounds are more easily detected when they follow a temporally predictable sequence. Isochrony is essential in individual interactions as temporal predictability supports cooperative engagement and enhances auditory detection [6].

Arousal is another critical influence on both speech and singing tempo in humans: high arousal is associated with an increase in speech rate [7], and songs have a faster syllable onset when expressing anger [8]. Similarly, animals can discriminate vocal expression of arousal [9], as it influences the timing of their vocalization. For example, marmoset monkeys vocalize faster during higher levels of arousal [10], and meerkats have shorter inter-call duration in high-urgency situations than in low-urgency ones [11]. Similarly, cape fur seals produce vocalizations with higher call rates during high-arousal-state interactions [12].

Recent evidence suggests that some temporal structures that characterize human music, specifically isochrony, are also found in animal singing behaviour [13–15]. Songs of non-human primates represent a promising model for understanding how the sense of rhythm originated in the primate phylogeny [16]. One way to understand why rhythmic vocal displays have evolved in primates is to conduct cross-species comparative research. Many of these displays may have parallels in the animal kingdom, particularly among non-human primates. According to Jordania [17], singing probably originated among our tree-dwelling ancestors. To date, singing has been observed in four primate families (Hylobatidae, Indriidae, Tarsiidae and Pitheciidae) that are not closely related, suggesting an independent evolution of song emission in the clade [18]. All small apes (Hylobatidae) considered so far show isochronous rhythms [19,20] except for the Hainan gibbon *Nomascus hainanus*, which shows two rhythmic categories [21]. Songs of the lemur *Indri indri* are even characterized by three different rhythmic categories [22,23]. It is unclear which factors have driven the emergence of different rhythmic organizations in the vocal behaviour of non-human primates. However, recent investigations suggested that the social context in which songs occur and the interactive nature of singing behaviour might play an important role [20,24].

However, to date, only eight species of singing primates have been studied, belonging to two of the four singing primate families (Hylobatidae and Indriidae) and representing approximately 11% of non-human primates exhibiting singing behaviour [16]. Therefore, it is still being determined whether the rhythmic features of non-human primates’ songs are conserved in the two yet-to-be-studied singing primate families, which comprise tarsiers and New World monkeys such as titi monkeys. Do these species sing with the same rhythmic structures of lemurs and lesser apes?

Coppery titi monkeys (*Plecturocebus cupreus*) are small arboreal primates inhabiting western Amazonian rainforests, where they live in small family groups composed of a monogamous pair and their offspring [24]. They exhibit long-term stable pair bonds and biparental infant care [25]. Coppery and other titi monkeys emit loud duets composed of identical sequences emitted by the two sexes but sung alternately and continuously cycle the different parts [26,27]. They use duets for joint resource defence and intergroup communication, which are sung both routinely in the early morning (‘advertisement songs’) and during intergroup encounters with neighbouring groups (‘territorial songs’; [28]). These features make titi monkeys a valuable model species to deepen our understanding of non-human primate singing behaviour’s rhythmic features and investigate social factors’ influence on duet timing. Would different social contexts, characterized by different arousal levels and urgency, impact the tempo and rhythm of titi monkeys’ duets?

We aim to investigate the presence of rhythmic categories in the loud duets of coppery titi monkeys, which has never, to our knowledge been tested before in any New World monkey. Specifically, we want to explore the link between social factors such as the context of duet emission, the presence of a newborn within the group and the rhythmic structure of duets. First, we predict that titi monkey duets would show an isochronous rhythm, in line with recent studies on singing primates [20–23], and second, that the singing tempo and the rate of temporal regularity will be influenced by the social factors considered, based on evidence on the effect of arousal on the structure of communicative signals.

## 2. Material and methods

### (a) Observations and recordings

The study took place at the Estación Biológica Quebrada Blanco in the Peruvian Amazon (4°21′ S, 73°09′ W) in October and November 2022. We recorded songs emitted by five pairs (10 adult individuals) of *P. cupreus* from five wild family groups that were habituated to human presence. Animal habituation took place between 1997 and 2017 depending on the group, and it took on average six weeks [28]. We recorded duets opportunistically from 4.00 to 13.00, as this is when animals usually vocalize [29]. We used a ZOOM F1 field recorder and a Lumix DCFZ-82 camera to record videos of the singing pairs. Since we were interested in understanding whether the temporal structure of songs depended on the context of emission, we noted the singers’ identity, the context in which duets were emitted, and the presence/absence of a newborn with the singing pair.

### (b) Acoustic analyses

We selected and saved in separate files all songs whose quality allowed subsequent quantitative analyses. An individual contribution comprises all the units emitted by a vocalizing animal. We obtained 162 individual contributions, 81 for males and 81 for females; 20 were sung at dawn (‘advertisement songs’) and 142 during intergroup encounters (‘territorial songs’). Advertisement songs are routinely given at dawn, acting as resource/mate defence [29] and given in the absence of direct visual contact. Territorial songs are given whenever two neighbouring groups are in visual contact (electronic supplementary material, table S1). Titi monkeys sang 114 contributions when a newborn was present and 48 with no newborn. Titi monkeys emitted, on average, only one advertisement song per day (1.1 ± 0.3) but 9 ± 5.8 territorial songs per day. We annotated the onsets and offsets of all the vocalizations emitted in each individual contribution to a song through the TextGrid tool in Praat (v. 6.2.05; [30]), identifying all vocal and non-vocal intervals. For each interval, we measured the starting point and its duration.

To evaluate the rhythmic structure of each individual contribution in titi monkeys’ duets, we calculated in RStudio [31]: (i) the inter-onset interval duration (hereafter *t*_k_), that is, the time between the onset of a note and that of the subsequent one [32]; and (ii) the ratio of two subsequent *t*_k_ (hereafter *r*_k_; electronic supplementary material, table S1). This operation involves dividing the duration of each *t*_k_ by the sum of its duration and that of the following one [13]. We obtained a total of 71 819 *t*_k_ values and 54 721 *r*_k_ values.

To evaluate the occurrence of small integer ratio, we divided the ratio distribution into on-integer and off-integer ratio ranges, centring the on-integer ratio range around 1 : 1 (or 0.50), 1 : 2 (or 0.33) and 2 : 1 (or 0,66). Following Roeske *et al*. [13], a 1 : 1 on-integer ratio is considered when *r*_k_ falls within the values between 0.444 and 0.555, and an off-integer ratio when *r*_k_ is between 0.4−0.444 and 0.555−0.6. The range of a 1 : 2 on-integer ratio is 0.308−0.364, and the off-integer ratio lies within 0.286–0.308 and 0.364−0.4. Lastly, for a 2 : 1 ratio, we consider an on-integer ratio within 0.636−0.692 and an off-integer ratio within 0.6−0.636 and 0.692−0.714. Next, we tallied all the ratios situated within the respective on-integer and off-integer ratio intervals for each individual contribution and corrected the count based on bin dimensions.

### (c) Statistical analysis

We created seven models (four generalized linear mixed models (GLMMs) and three linear mixed models (LMMs)) to investigate the factors affecting the temporal structure of titi monkeys’ songs. For each ‘full model’, consisting of response, fixed and random factors, we tested the significance against a ‘null model’ that included only random factors, using a likelihood ratio test (analysis of variance with the ‘Chisq’ argument; [33]). For models that significantly differed from null ones, we obtained model results via *summary* function and proceeded with post hoc tests (*emmeans* package; [34]) to compare all possible pairwise combinations among the fixed factor levels for all models with a *p*-value adjustment based on the Tukey method. In the following sections, details of the models’ syntax are provided.

#### (i) Rhythmic categories and regularity

We used four GLMMs (*glmmTMB* package; [35] to investigate the presence of rhythmic categories in titi monkey duets and their rhythmic regularity (electronic supplementary material, table S1). Before creating the models, we checked the distribution of the response variable with the package *fitdistrplus* [36], resulting in *beta* as a suitable theoretical distribution:

- *distribution of rhythmic categories:* in the first model, we used the context in which duets took place (advertisement/territorial) in interaction with the *r*_k_ bin type (factor levels: OFF1 : 1, ON1 : 1, OFF1 : 2, ON1 : 2, OFF2 : 1, ON2 : 1) as fixed factors. In the second model, we used the sex of the vocalizing animal in interaction with the *r*_k_ bin type, with the same factor levels as the first model as fixed factors. For both models, we used the individual ID and individual contribution ID to a duet as nested random factors. The comparison with the null model was significant for both models ($\chi_{11}^{2}$= −4315.593, *p* < 0.001 and $\chi_{11}^{2}$= −4163.430, *p* < 0.001, respectively). We then ran a post hoc test for multiple comparisons between the observations falling in each bin type and, in the first case, the context of emission, while in the second case, the caller’s sex; and
- *isochrony rate*: we calculated the regularity rate for the isochrony peak as the ratio between the count of on-integer observation and on-plus off-integer observations of the individual contribution. We used the context of emission and the interaction between the sex of the emitter and the presence/absence of infants within the pair as fixed factors. The individual ID was used as a random factor. For this first model, the comparison between the full and null models was not significant ($\chi_{4}^{2}$= 4.728, *p* = 0.316). Therefore, we investigated whether the variation of the isochrony rate was better explained by individual variability, and we ran a second model where the individual ID, the context of emission and the presence/absence of an infant were fixed factors. We used group ID as a random factor in this second model. In this case, the null model significantly differed from the null one ($\chi_{11}^{2}$= −456.001, *p* = 0.002). We then performed pairwise comparisons for each level of the explanatory variable (ID_ind) with a post hoc test.

#### (ii) *t*_k_ and silent gap duration

To investigate the distribution of *t*_k_’s duration and the silent gap between notes, we used three LMMs (*lme4* package; [37]), where the variable durations were logarithmically transformed:

- *t*_k_ *duration:* we included the log-transformed *t*_k_ durations as the response variable, the context of emission and the interaction between the sex of the emitter and the presence/absence of infants within the pair as fixed factors. The individual ID and contribution ID to a duet were used as nested random factors. The full model significantly differed from the null one ($\chi_{4}^{2}$= 15.101, *p* = 0.004). We compared all the possible comparisons among the levels of the interacting variables (sex and presence/absence of an infant) with a post hoc test;
- *t*_k_ *duration and rhythmic regularity:* we calculated the log-transformed mean value of *t*_k_ for each individual contribution, which was used as the response variable. We used as the fixed factor the rate of regularity of each individual contribution, and the individual ID as a random factor. The full model significantly differed from the null one ($\chi_{1}^{2}$= 16.31802, *p* < 0.001); and
- *silent gaps duration*: we used the duration of silent gaps between notes as the response variable and the context of emission and the interaction between the sex of the emitter and the presence/absence of infants within the pair as fixed factors. The individual ID and contribution ID to a duet were used as nested random factors. The full model significantly differed from the null one ($\chi_{4}^{2}$= 17.227, *p* = 0.002). We compared all the possible comparisons among the levels of the interacting variables with a post hoc test.

## 3. Results

### (a) Distribution of rhythmic categories: titi monkeys sing isochronous songs

**Figure 1.**
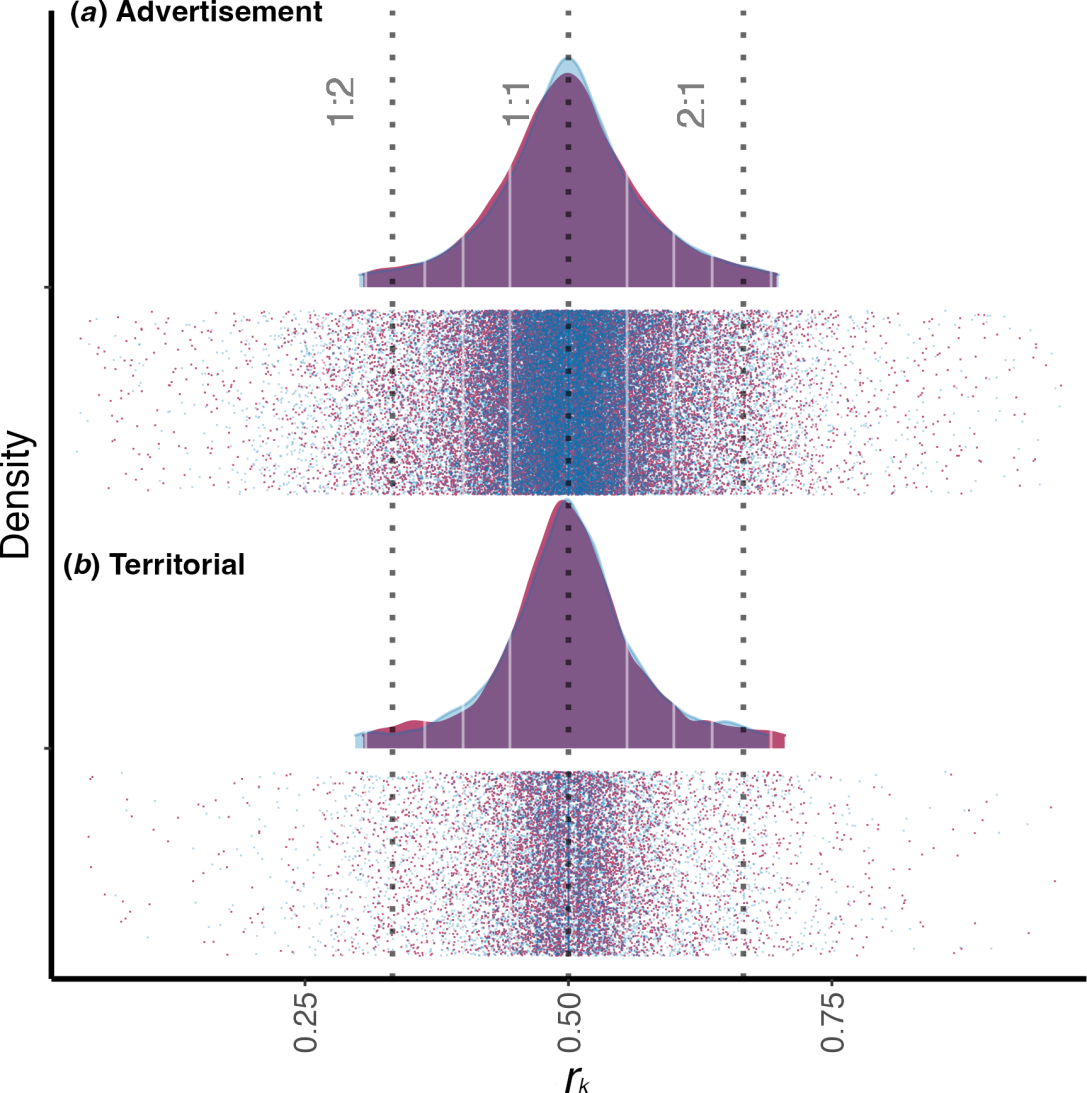
Probability density functions of rhythm ratios (*r*_k_) in the two contexts of emission for males (light blue) and females (purple) individuals. (*a*) Advertisement, (*b*) territorial. A peak around 0.5 ratio represents isochrony. White lines correspond to on- and off-integer ratio ranges. Dotted lines correspond to ratio values. Dots below the density plots represent raw values.

Density plots of *r*_k_ values revealed a single cluster that aligned with isochronous ratios (1 : 1 ratio; figure 1). Post hoc comparisons showed that apparent isochronous components characterize titi monkeys’ songs but do not possess a 1 : 2 and 2 : 1 ratio, in both contexts of emissions (1 : 1 ratio, *p* < 0.001; electronic supplementary material, table S2). Both duets emitted in the advertisement context and duets emitted in the territorial one showed a single, significant peak around isochronous values. Moreover, males and females showed a single, significant peak at 1 : 1 isochrony (males = OFF1 : 1-ON1 : 1, *p* < 0.001; females = OFF1 : 1-ON1 : 1, *p* < 0.001; electronic supplementary material, table S2).

### (b) Rhythmic regularity: individuals differ in isochrony rate and songs sung when an infant is present are less regular

**Figure 2.**
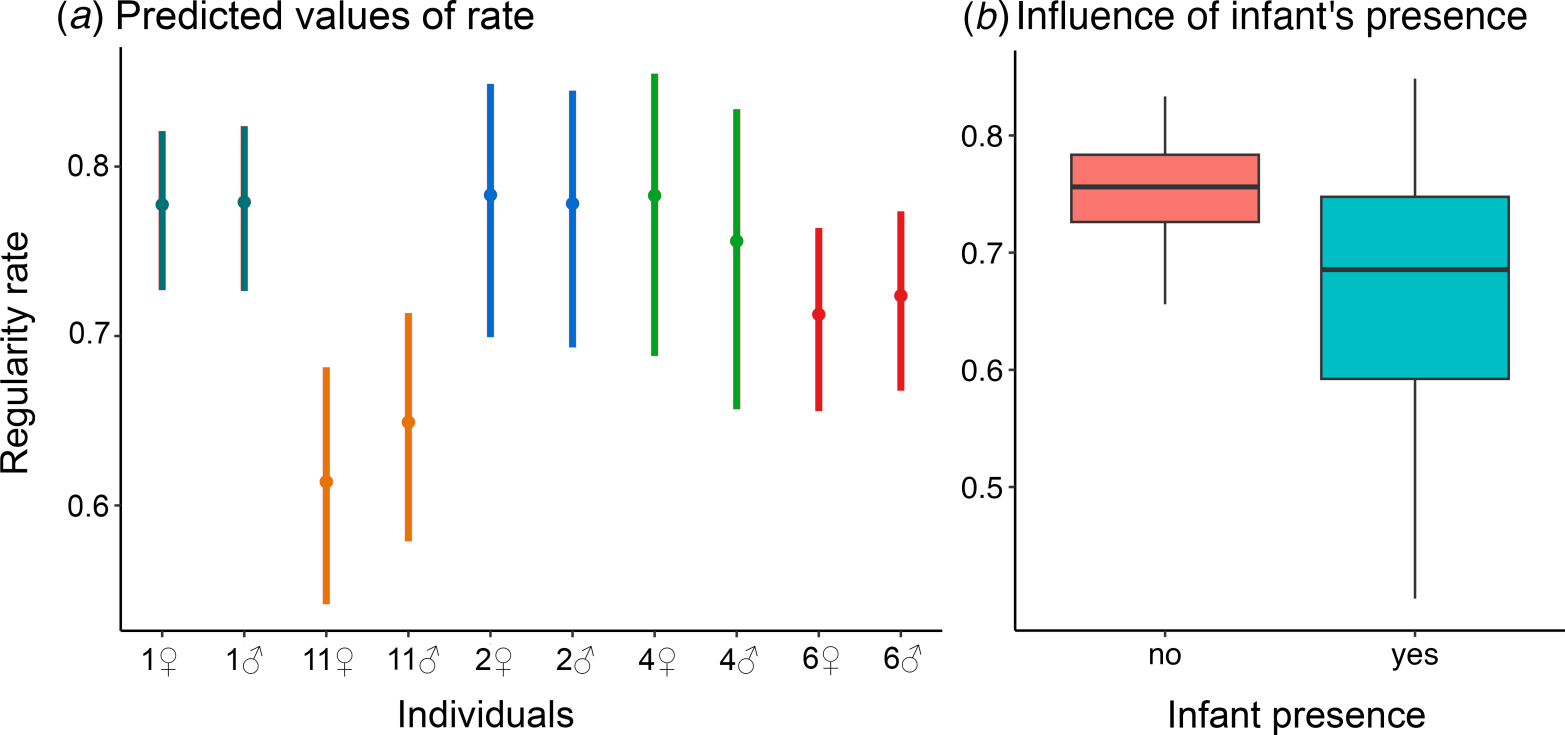
(*a*) Marginal effects plot of individual differences in regularity rate. Central dots represent predicted values, with solid bars as confidence intervals. Colours represent different groups. (*b*) Boxplots showing the influence of the presence of an infant on the rate of regularity of titi monkeys’ duets. Horizontal lines represent the median values; whiskers indicate value ranges.

We found an effect of infant presence on the rhythm regularity, with songs being more regular in the absence of an infant (*p* = 0.044; electronic supplementary material, table S3; figure 2), while the context of song emission did not influence the regularity of titi songs (*p* = 0.089; electronic supplementary material, table S3; figure 2). Moreover, our model showed that the rate of isochrony in titis duets differed between individuals: the male and the female of group 11 were less regular than the individuals of group 1 (*p* < 0.001 for each comparison; electronic supplementary material, table S3; figure 2), and the same was true also for group 2, where the female of group 2 differed from the one of group 11 (*p* < 0.001), as well as from the male of group 2 (*p* = 0.001), and the male of group 11 differed from that of group 2 (*p* = 0.016) and the female of group 2 (*p* = 0.026). Finally, group 11 also partially differed in rhythmic regularity from individuals of group 4: the female of group 11 was less regular than the female (*p* = 0.006) and the male (*p* = 0.050) of group 4, while the male of group 11 did not differ from the one of group 4 (*p* = 0.302) and showed only a tendency from the female of the same group (*p* = 0.062). None of the other comparisons were significant, and, in particular, members of the same pair did not show significant differences in terms of regularity rate (electronic supplementary material, table S3).

### (c) *t*_k_ duration: songs are faster when an infant is present, with no differences between sexes

**Figure 3.**
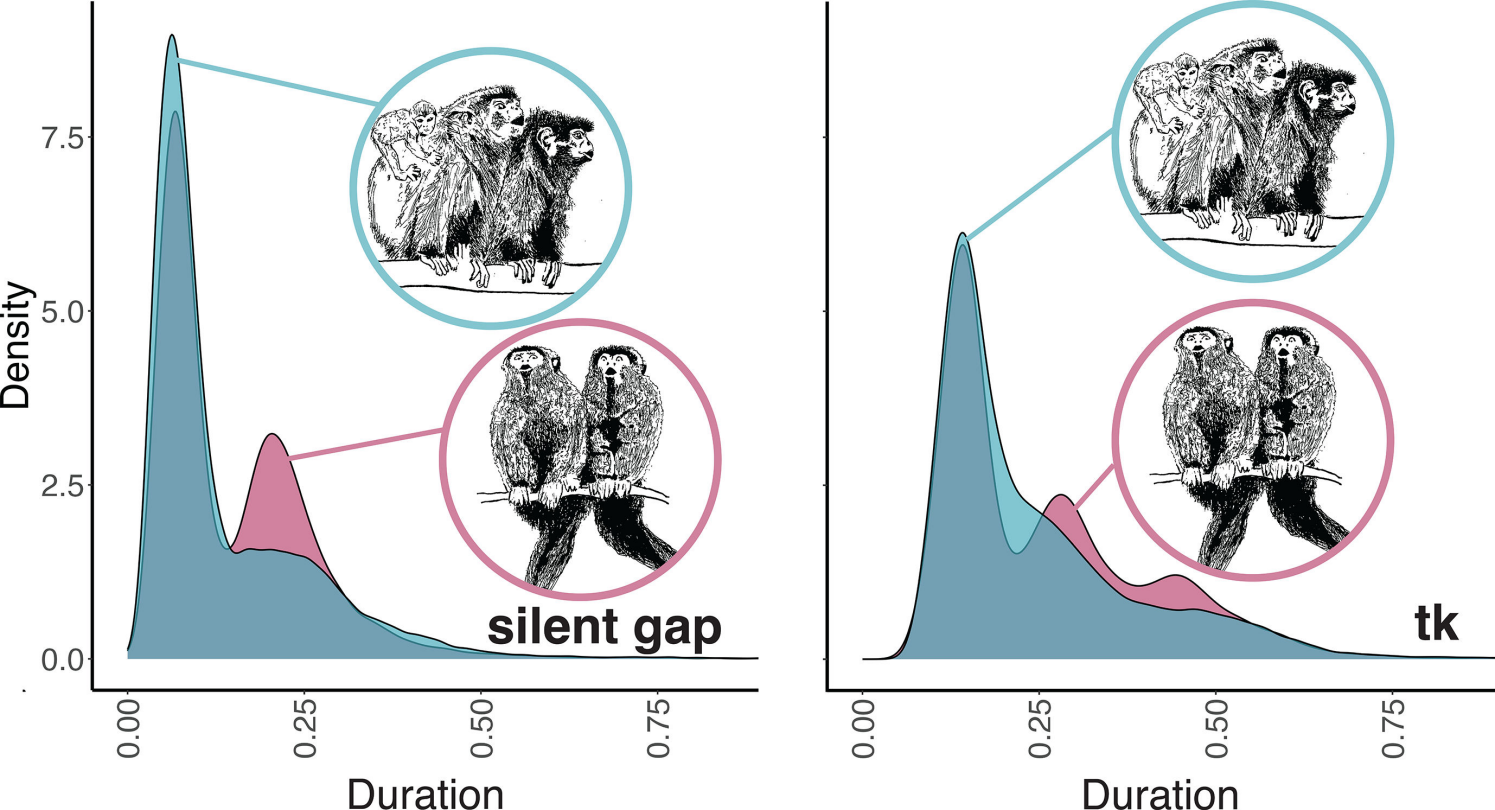
Probability density functions of *t*_k_ and silent gaps duration. (a) *t*_k_ duration when infants are present (light blue) and when they are not (light purple). (b) Silent gaps duration between notes emitted when infants are present (light blue) and when they are not (light purple). Drawing in the circles represents the two social contexts.

*t*_k_ showed a different distribution depending on the social context. In particular, songs emitted when an infant was not present showed a multimodal distribution with three peaks (at 0.141 s, 0.282 s and 0.439 s), while they had an unimodal distribution with a peak at 0.139 s (figure 3) when there were no infants within the group. Titi monkeys sang faster when an infant was present in the group (*p* = 0.001; electronic supplementary material, table S4), being those songs characterized by shorter *t*_k_ (figure 3). On the other hand, songs given in the territorial context had longer *t*_k_ than those emitted in the advertisement ones and, thus, were sung at a slower pace (*p* = 0.019; electronic supplementary material, table S4). We found no differences in overall *t*_k_ duration between males and females (*p* = 0.066; electronic supplementary material, table S4). However, the post hoc comparison showed that females sang faster when infants were present (*p* = 0.007; electronic supplementary material, table S4).

### (d) Relationship between *t*_k_ duration and rhythmic regularity: faster songs are more regular

We found that the mean tempo value for each individual contribution was positively correlated with the regularity rate, namely that faster tempos were associated with a higher rate of regularity (estimate = 0.7552, *t*_144.722_-value = 4.1679, *p* < 0.001).

### (e) Silent gaps duration

Gap duration showed a different distribution depending on the social context, with a bimodal distribution for songs emitted in the absence of infants (peaks at 0.065 s and 0.209 s; figure 3) and a unimodal distribution for songs emitted when infants are present (peak at 0.634 s). Songs emitted when an infant is present in the group have shorter gap durations independently of the context of emission (*p* < 0.00; electronic supplementary material, table S5). Differently, songs given in the territorial context have longer silent gaps between notes than advertisement songs (*p* = 0.004; electronic supplementary material, table S5). Males and females did not show overall differences in silent gap duration (*p* = 0.166), but the presence of an infant shortened the gap duration of females but not of males (*p* = 0.004; electronic supplementary material, table S5).

## 4. Discussion

Our study shows that titi monkeys’ songs are fundamentally isochronous, regardless of the emitter’s sex and the context in which singing behaviour takes place. Nevertheless, the temporal structure of their song is not entirely fixed, as its regularity and tempo depend on the social context in which animals vocalize. Our results also indicate individual differences in how precisely the animals match the isochronous rhythm.

### (a) Isochrony is a feature of the songs of the coppery titi monkey and it facilitates coordination

The research findings indicate that isochrony is a prominent rhythmic characteristic of *P. cupreus*’s song. This result is consistent with previous studies on other singing primates [19–23] and strengthens the link between the rhythm of non-human primate songs and that of human music [17]. It also aligns with the hypothesis that beat-based timing is an important feature of the primate lineage [18]. A strong isochronous rhythm was present in the individual contributions of both male and female coppery titi monkeys, and both contexts of emission were considered. Both songs—the ones given routinely at dawn and those emitted during territorial confrontations—have the same regular rhythm. Similarly, songs emitted by *Indris* in different contexts of emission showed a strong isochronous rhythm despite differing in terms of other categorical rhythms [22].

The prevalence of isochrony in primate long-distance communication is probably owing to the many benefits that isochronous signals have: first, isochrony helps maximize signal redundancy and minimize its entropy. Second, it plays an important role in group coordination and synchronization, not only in humans [38,39] but also in other singing apes: white-handed gibbons are more isochronous when performing duet interaction than when singing alone [20], and the regularity of *Nomascus* spp. male songs facilitates the synchronization of females' great calls [21]. In line with this evidence, we found that titi monkey pair-mates did not differ in terms of isochrony rate. In other words, individuals can be more or less precise in matching the isochronous rhythm, but the pair members will perform isochronous songs with the same degree of precision. We thus hypothesize that the observed isochrony in the singing of coppery titi monkeys plays a role in boosting pair synchronization and coordination as it does in other singing primates.

Interestingly, the pair that showed less regularity consisted of a relatively recent couple formed in 2018 [24], while all others existed for longer periods. Our results align with previous work showing that pair-mates of this species show vocal convergence [40] as the isochronous rhythm could be one of the factors that guide the pair members of a pair in having the same rate of note emission. Moreover, the differences we observed in the rhythmic regularity among individuals align with previous findings on individual features that characterize specific parts of the titi monkeys’ song [41].

### (b) Social influences on singing tempo and regularity

Our prediction regarding the influence of arousal/agitation on the singing tempo was only partially supported. While duets emitted when an infant was present were generally faster than when there was not an infant, those emitted during intergroup encounters at the territory border were overall sung at a slower pace than those emitted routinely at dawn. In birds, changes in singing rate can be informative about the changes in motivation during territorial context, with more aggressive responses towards faster playback stimuli [42]. So why do titi monkeys seem to sing with a slower *tempo* during territorial confrontations? A possible explanation is that the slower rate of note emission of territorial duets could be partly influenced by the fact that titi monkeys sang, on average, 8.9 songs during each intergroup encounter. At the same time, they advertised their presence in their territory by singing once a day at dawn. Previous work highlighted an effect of age on the rate of note emission in titi monkeys, probably owing to difficulty in quickly repeating song elements [40]. Therefore, we might expect that a possible effect of arousal on the singing tempo can be somehow masked by the energetic costs associated with an extended singing time. This explanation also aligns with our findings on territorial songs having longer silent gap durations than the ones given in the advertisement context, as the slower tempo results from taking longer pauses between notes rather than emitting longer notes.

On the other hand, it is known that the presence of infants can influence non-human primates’ behaviour and social interactions [43] and in particular, that parents can discriminate between the risk for themselves and that for their infants [44,45]. The presence of infants plays a role in the behaviours of coppery titi monkey pairs, with couples without offspring displaying more affiliation than couples that have them [46]. The infants present in the groups at the time of our data collection ranged from a few days to a couple of months old, being particularly vulnerable. Therefore, we suggest that their presence can influence parents’ duets, which are used for joint resource defence and intergroup communication. Indeed, the ownership of a territory and its resources is critical for infant survival and parents’ fitness.

We found that the presence of infants impacted not only the singing *tempo* but also its regularity: while, overall, there was a positive correlation between tempo and regularity, titi pair-mates sang less regular songs when an infant was present. Fedurek *et al.* [47] investigated trade-offs in chimpanzees’ vocalizations and concluded that they could be owing to biomechanical constraints on the fast production of vocal sequences. Also, barks of cape fur seals were less rhythmically precise when emitted at higher rates [48]. Similarly, there is evidence in humans that speech production can be constrained by a speed-accuracy trade-off, although with some variability across subjects [49]. In our case, this trade-off seems to be present only in particular contexts, i.e. situations in which vocalizing might influence the parents’ fitness.

A second aspect is that contrary to what happens during pregnancy, coppery titi monkeys show a decline in pair affiliation during the postpartum period [24,50]. This reduced affiliation could also be reflected in the reduced regularity of singing. Also, lactation is more energetically costly than pregnancy [51,52] and this could impact costly behaviour, such as the emission of a loud series of fast notes.

The presence of infants, besides affecting the communicative signal of the groups in which newborns are present, can also impact the receivers of the message, in this case, neighbouring titi monkey groups. As suggested by Scott-Phillips *et al.* [53] the message carried by an isochronous vocalization is its ‘isochronicity,’ and therefore, the regularity and tempo of titis’ duets may also affect the arousal levels of their conspecifics, which could increase their alertness in certain situations. In humans, music with a fast tempo can significantly impact motivation and physiology during exercise [54,55]. Listening to music can also increase heart rate during exercise [56]. Similarly, in coppery titi monkeys, those individuals who sing at a faster tempo may have a greater influence on the arousal levels of conspecifics participating in singing behaviour.

In conclusion, our work provides new evidence regarding the presence of an isochronous rhythm in the songs of a New Word monkey, expanding the investigation of categorical rhythm beyond lemurs and apes. We also explored the proximate causes of titi monkeys rhythm, as we not only highlighted the presence of potential costs and constraints on the production of primate duets but also linked them to social factors that can shape their timing and regularity.

## Data Availability

Data and R code used for this study can be accessed at [57]. Supplementary material is available online [58].
